# Comparing the Characteristics of Cigarette Smoking and e-Cigarette and IQOS Use among Adolescents in Taiwan

**DOI:** 10.1155/2020/7391587

**Published:** 2020-06-20

**Authors:** Yue-Chune Lee, Li-Chuan Chang, Chieh Hsu, Pei-Ching Chen

**Affiliations:** ^1^Institute of Health and Welfare Policy, School of Medicine, National Yang-Ming University, Taipei City 112, Taiwan; ^2^Master Program on Trans-Disciplinary Long-Term Care and Management, National Yang-Ming University, Taipei City 112, Taiwan; ^3^Department of Health and Welfare, College of City Management, University of Taipei, Taipei City 111, Taiwan

## Abstract

**Objectives:**

Our objective in this study was to identify the risk factors for cigarette, e-cigarette, and IQOS use among adolescents in Taiwan, with a particular focus on socioeconomic status, smoking status of parents and peers, cigarette promotions, and anti-tobacco campaigns.

**Methods:**

Data were obtained from the 2018 version of the annual cross-sectional Taiwan Global Youth Tobacco Survey, which is used to monitor tobacco use among Taiwanese adolescents in junior and senior high schools. The dependent variables in the study were “current cigarette smoking,” “current use of e-cigarettes,” and “current use of IQOS devices” (i.e., during the 30 days prior to survey completion). Independent variables included gender, school grade, monthly income/allowance, educational level of parents, smoking status of parents, smoking status of close friends, access to free cigarettes, exposure to cigarette advertisements, and attendance at anti-tobacco courses. Logistic regression was used in the identification of factors correlated with the current use of cigarettes, e-cigarettes, or IQOS.

**Results:**

We determined that 5.65% of the adolescents in the study were currently using cigarettes, 2.74% were currently using e-cigarettes, and 2.33% were currently using IQOS. Our analysis revealed a number of factors that have a bearing on smoking behavior, including gender, monthly allowance, educational level of parents, smoking status of parents and close friends, access to free cigarettes, and exposure to cigarette advertisements.

**Conclusions:**

The tobacco product that was most widely used by adolescents was cigarettes, followed by e-cigarettes and IQOS. The socioeconomic status, smoking status of parents/close friends, and access to cigarettes were all identified as important factors related to the current use of cigarettes, e-cigarettes, and IQOS by adolescents.

## 1. Introduction

Heated tobacco products (HTPs) are similar to e-cigarettes in their use of heat to volatilize nicotine (in a nicotine-containing liquid) to just below the point of combustion, so that users inhale aerosols rather than smoke. “I-Quit-Ordinary-Smoking” (IQOS) is a typical HTP created by Philip Morris International [1] to resemble conventional cigarettes [2].

The focus of HTP marketing on young people [3] has led to a significant increase in the prevalence of e-cigarette and IQOS use among adolescents around the world [2, 4–9]. Cullen et al. [10] reported an increase in current e-cigarette use among high school students in the United States (US) from 1.5% in 2011 (220,000 students) to 20.8% in 2018 (3.05 million students). They also reported an increase in current e-cigarette use among US middle school students from 0.6% in 2011 (60,000 students) to 4.9% in 2018 (570,000 students). Osibogun et al. [11] reported that current e-cigarette use among US youth is associated with higher odds of transitioning to regular cigarette smoking.

A number of studies have explored the risk factors associated with the use of e-cigarettes among adolescents [7, 12–16]. One study [7] recently reported an increase in the ever-use of e-cigarettes among adolescents self-described as never-smokers. Researchers have also reported that adolescents who use e-cigarettes are significantly more likely than their nonsmoking counterparts to engage in substance abuse [12]. Due, in part, to the gradual increase in IQOS use among adolescents, researchers have yet to elucidate the associated risk factors or conduct comparisons with the risk factors of other tobacco products. At present, the use of e-cigarettes in Taiwan is illegal, and the government is actively amending the law to prevent the use of other HTPs. Nonetheless, there are many channels through which young people are able to gain access to e-cigarettes and other HTPs. In 2018, the Taiwan Global Youth Tobacco Survey (GYTS) first investigated the issue of HTPS. Based on the data obtained in that survey, this is the first study to examine the risk factors for cigarette, e-cigarette, and IQOS use among adolescents.

Wu et al. [17] reported that higher socioeconomic status was associated with the use and intention to use HTPs. Rennie et al. [15] reported that higher socioeconomic status positively predicted the likelihood of experimenting with e-cigarettes and cigarettes. Thus, our objective in this study was to identify the risk factors of cigarette, e-cigarette, and IQOS use among adolescents, with a particular focus on the socioeconomic status as well as the smoking status of key persons (close friends and family members), access to free cigarettes, effectiveness of promotions, and anti-tobacco campaigns in Taiwan.

## 2. Materials and Methods

### 2.1. Data Sources

This study was based on data obtained from the Taiwan GYTS 2018, which has been conducted annually in junior and senior high schools by the Health Promotion Administration (HPA) of Taiwan since 2010. This survey is meant to facilitate the statistical analysis of tobacco use patterns among adolescents in Taiwan and other countries [18]. This self-administered questionnaire is presented in the classroom, and the anonymity of participants is strictly observed [19]. The survey has incorporated questions pertaining to the use of e-cigarettes since 2014 and IQOS since 2018.

Local health bureaus oversee the administration of the survey after the staff undergoes training by the HPA. This paper survey is administered in all classrooms simultaneously to prevent students discussing the questions beforehand. The HPA submitted this investigation to the Institutional Review Board for review. In accordance with laws pertaining to human research performed by legal authorities, the survey was exempted from the need to obtain consent from respondents. Prior to the survey, the students and their parents were informed of the survey objectives as well as the right of the students not to participate [14].

### 2.2. Study Population

Details pertaining to sampling sizes and response rates on the Taiwan Global Youth Tobacco Survey have not been disclosed by the HPA. They have disclosed only the sampling rules to be observed by the county or city in recruiting 900 students from grades 7–9 and 1100 students from grades 10–12. Sampling involved selecting the school first, then selecting classes in that school, and finally the students.

**Figure 1:**
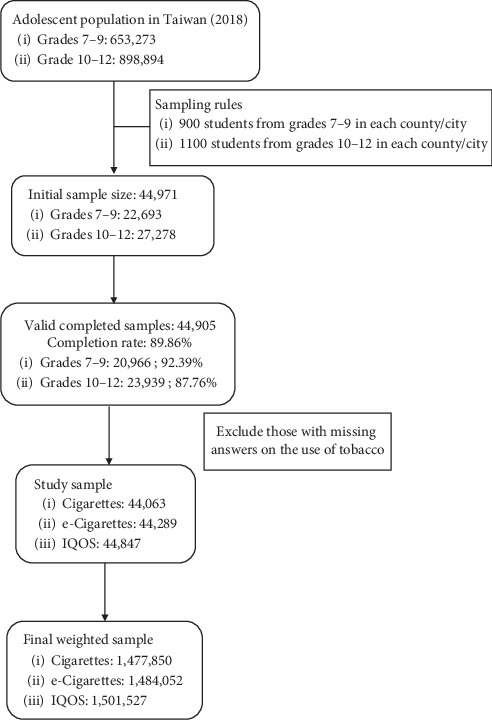
Flow diagram illustrating process by which study sample was assembled.

The 2018 survey included 49,971 students throughout Taiwan, comprising 22,693 students in junior high and 27,278 students in senior high. That survey yielded an overall completion rate of 89.86% (44,905 valid responses): junior high school (92.39%; 20,966) and senior high school (87.76%; 23,939) [20]. Samples missing answers pertaining to previous cigarette smoking and the previous use of e-cigarettes or IQOS were excluded. The number of students who answered questions related to tobacco use was as follows: cigarettes (44,063), e-cigarettes (44,289), and IQOS (44,847). Sample weighting was used to ensure that the data were representative of adolescents (grades 7 to 12) in Taiwan. After sample weighting, the number of adolescents in Taiwan represented in this survey was estimated as follows: cigarettes (1,477,850), e-cigarettes (1,484,052), and IQOS (1,501,527).  Figure 1 presents a flow diagram illustrating the process by which the study sample was assembled.

### 2.3. Variables

The dependent variables in the study were “current cigarette smoking,” “current use of e-cigarettes,” and “current use of IQOS devices.” Note that current use refers to the 30 days prior to completion of the survey. Current cigarette smoking was indicated by a positive response to the question, “have you consumed any cigarettes in the past 30 days? (yes/no).” Current use of e-cigarettes was indicated by a positive response to the question, “have you used any e-cigarettes in the past 30 days? (yes/no).” Current use of IQOS was indicated by a positive response to the question, “have you used an IQOS device in the past 30 days? (yes/no).”

The independent variables included gender, school grade (7–12), smoking status of key persons, socioeconomic status, exposure to cigarette advertisements, access to free cigarettes, and participation in anti-tobacco courses.

The smoking status of key persons was derived from the smoking status of parents (both parents smoke, only the father smokes, only the mother smokes, and neither smokes) and close friends (binary choice: yes/no).

Socioeconomic status was estimated in terms of the educational level of parents (junior high school and below, senior high school, university or college, and graduate school) as well as the monthly income/allowance of the student (US$0, US$0.03–49.71, US$49.72–116.03, and US$ ≧ 116.04). Note that the conversion rate of NT dollars to US dollars was US$1 = NT$30.156 in 2018. We adopted the higher education level between the two parents, and in cases where one value was missing, we used the obtained value.

Questions about exposure to cigarette advertisements could prompt three possible responses, reporting (1) “exposure to cigarette advertisements in retail outlets,” (2) “no exposure to cigarette advertisements in retail outlets,” and (3) “did not visit any retail outlets.” The dependent variable is subject to the possible responses to the original question, which are not mutually exclusive. Thus, we merged the responses into two categories. One category involved reporting exposure to cigarette advertisements, whereas the other involved reporting no exposure to cigarette advertisements and not visiting any retail outlets.

Access to free tobacco products was indicated by a positive response to the question “have you ever been offered a free tobacco product by a tobacco company? (yes/no).” Exposure to anti-tobacco courses was indicated by a positive response to the question “have you been instructed about the dangers of tobacco use in the past 12 months? (yes/no).”

### 2.4. Analysis

The Rao-Scott chi-square test was used to elucidate the association between the independent variables (demographic characteristics) and the dependent variable (use of cigarettes/e-cigarettes/IQOS), which were adjusted in accordance with the sample design (i.e., county/city and school). Logistic regression was also adjusted in accordance with the sample design (i.e., county/city and school) to identify factors indicating a correlation with the current use of cigarettes, e-cigarettes, and IQOS. All analyses were performed using SAS software version 9.4 (SAS Institute Inc., Cary, NC, USA), and *P* < 0.05 was used to define statistically significant results.

## 3. Results

A significant percentage of the respondents reported using cigarettes (5.65%), e-cigarettes (2.74%), or IQOS devices (2.33%) within the 30 days prior to the survey. When extrapolated to the entire country, this represents an estimated number of 83,480 using cigarettes, 40,723 using e-cigarettes, and 35,011 using IQOS devices.

Despite differences in the number of students who responded to the three variables, the overall distribution of characteristics among the surveyed adolescents varied only slightly. Most of the adolescents were boys, in senior high school, and had a low monthly allowance. Most of the adolescents in this study had parents with a middle education level, most of their parents did not smoke, and most of their close friends did not smoke either. Most of the adolescent had not received free cigarettes, most had not been exposed to cigarette advertisements, and most had received instruction concerning the dangers of tobacco (see Table 1).

In terms of demographic characteristics, we observed significant differences between current smokers and nonsmokers, regardless of the tobacco products. Compared to non-smokers, all of the groups of smokers (cigarettes, e-cigarettes, and IQOS devices) included a higher percentage of boys, a lower percentage in junior high school, and a lower percentage with a small monthly allowance. The smokers also included a higher percentage of parents with a low education and a higher percentage of parents or close friends who smoke. Finally, a higher percentage of the smokers reported having access to free cigarettes and exposure to cigarette promotions, whereas a lower percentage of the smokers reported having attended anti-tobacco classes. Note that a lower percentage of the IQOS users reported having attended anti-tobacco classes; however, the difference did not reach the level of significance (see Tables 1–3).

Table 4 lists the factors correlated with higher odds of tobacco use. Note that the factors varied slightly with the type of tobacco product (cigarette, e-cigarette, or IQOS). Most of the tobacco users were boys, in senior high school (cigarette smoking only), with a middle/high monthly allowance, at least one parent who smokes, and close friends who smoke. Most of the tobacco users also had access to free cigarettes and had been exposed to cigarette advertisements (cigarette smoking and e-cigarette use only). The factors correlated with lower odds of tobacco use varied slightly. It appears that as grade level increased, the odds of IQOS use decreased and a low monthly allowance had the same effect. The higher the education level of the parents is, the less likely the adolescents were to smoke cigarettes, while attending classes on the dangers of tobacco lowered the odds of both cigarette smoking and e-cigarette use.

## 4. Discussion

Using data obtained in 2018, we determined that 5.65% of the adolescents in Taiwan were currently using cigarettes, 2.74% were currently using e-cigarettes, and 2.33% were currently using IQOS. Thus, the prevalence of cigarette smoking among adolescents is still higher than that of e-cigarette and HTP use. Nonetheless, it appears that the prevalence of cigarette smoking has decreased from the levels observed in 2016 (6.6%) [14]. The mutable factors associated with cigarette smoking include exposure to free cigarettes, anti-tobacco courses, and cigarette advertising. Thus, we recommended that governments continue bans on the marketing and advertising of cigarettes and that schools continue in their efforts to teach students about the hazards of smoking.

The prevalence of current IQOS users in the current study (2.33%) is consistent with the findings of Kim et al. [9], who reported, in 2017, that 3.5% of young Korean adults (aged 19–24 years) were using IQOS devices. The slight discrepancies between these values can probably be attributed to differences in the study methods, that is, our school-based survey and Kim's use of an online survey. Furthermore, the subjects in our study were adolescents (12–18 years), whereas Kim's study focused on young adults (19–24 years).

We identified correlations between socioeconomic status and the use of cigarettes, e-cigarettes, and IOQS. Adolescents with a higher monthly allowance were more likely to use cigarettes and e-cigarettes, whereas adolescents with a low monthly allowance were less likely to use IQOS. This is consistent with the findings of White et al. [5], who reported that adolescents with a higher monthly allowance were more likely to having ever used e-cigarettes. Adolescents with well-educated parents were less likely to use cigarettes, e-cigarettes, and IQOS. This is consistent with the findings of Kinnunen et al. [7], who reported that adolescents with less-educated parents were more likely to use e-cigarettes on a weekly basis, and Thrasher et al. [13], who reported that adolescents with well-educated parents were less likely to having ever used e-cigarettes.

We also observed correlations between the smoking status of key persons and the use of cigarettes, e-cigarettes, and IQOS. Adolescents with both parents or only one parent who smoked were more likely to use cigarettes and e-cigarettes, whereas adolescents who had only the father who smoked were less likely to use IQOS. This is consistent with the findings of Kinnunen et al. [7], who reported that adolescents whose both parents smoked or only one parent smoked were more likely to use e-cigarettes on a weekly basis. Moreover, adolescents who had close friends who smoked were more likely to use cigarettes, e-cigarettes, or IQOS. This is consistent with the findings of White et al. [5] and Thrasher et al. [13], in which it was reported that adolescents who had close friends who smoked were more likely to having ever used e-cigarettes.

Finally, we observed correlations between cigarette promotions and the use of cigarettes, e-cigarettes, and IQOS. Adolescents who had access to free cigarettes were more likely to use cigarettes, e-cigarettes, and/or IQOS. Adolescents who had been exposed to cigarette advertisements were more likely to use cigarettes, e-cigarettes, and IQOS, although the results related to IQOS use did not reach the level of significance. This is consistent with the findings of Kinnunen et al. [7], in which it was reported that adolescents who had seen e-cigarette advertisements were more likely to use e-cigarettes on a weekly basis. We suspect that this can be attributed to the fact that IQOS users were swayed by cigarette advertisements.

Furthermore, adolescents who had attended classes concerning the dangers of tobacco were less likely to use cigarettes and/or e-cigarettes; however, this was not shown to affect the prevalence of IQOS use. We suspect that this can be attributed to the fact that most courses focus on the hazards of tobacco and do not address the risks of IQOS. We therefore recommend that schools include the issue of IQOS in classes on the dangers of tobacco.

## 5. Limitations

This study had a number of limitations, such that our findings must be interpreted with caution. First, we used cross-sectional data, which means that we presented the factors correlating with the use of cigarettes, e-cigarettes, and IQOS. Future researchers could conduct follow-up investigations into the causal relationships between risk factors and the use of these three nicotine products. Second, the fact that this questionnaire was self-administered meant that we were unable to verify the actual smoking behavior of the subjects, with the result that the prevalence of cigarettes, e-cigarettes, or IQOS use may have been underestimated or overestimated. Third, this study did not assess variables related to the tobacco products themselves, such as the type or cost of the e-cigarette or IQOS. Finally, adolescents who were not enrolled in school at the time of the survey were excluded from analysis. Future researchers could seek to elucidate this.

The regulations pertaining to HTPs in Taiwan are somewhat unclear. Currently, e-cigarette products are not listed openly (i.e., electronic cigarettes are illegal). According to Article 14 of the Tobacco Hazards Prevention Act, “no person shall manufacture, import, or sell candies, snacks, toys, or any other objects in form of tobacco products.” e-Cigarettes that contain nicotine are treated as a drug (i.e., a violation of the Pharmaceutical Affairs Act). Nonetheless, HTPs and e-cigarette products are officially illegal. The Tobacco Hazards Prevention Act has not been amended since it was passed on January 11, 2009; however, suggested amendments banning the manufacture, importation, sale, and advertising of HTPs and e-cigarettes have recently been discussed. Due to the prevalence of HTP use among the youth, we recommend that the government propose amendments to “The Tobacco Hazards Prevention Act” pertaining to the control of e-cigarettes and HTPs as soon as possible.

## 6. Conclusion

Our results revealed that the socioeconomic status, smoking status of key persons, and access to cigarettes are the most important factors related to the current use of cigarettes, e-cigarettes, and IQOS by adolescents. The factor with the highest predictive value for the use of each tobacco product was the smoking status of close friends. Note, however, that, in addressing this issue, policy decisions are limited to creating a smoke-free campus. We, therefore, recommend that government adopt amendments to “The Tobacco Hazards Prevention Act” pertaining to the control of e-cigarettes and HTPs. Governments should also continue promoting smoke-free campuses and funding for courses on the hazards of tobacco (with a particular focus on e-cigarettes and IQOS) and impose controls on the advertising and promotion of cigarettes, e-cigarettes, and IQOS.

## Figures and Tables

**Table 1:** Demographics of cigarette smokers and non-smokers among Taiwanese adolescents in 2018.

Characteristic	All (*N* = 1477850)				Cigarette non-smokers (*n* = 1394370)			Cigarette smokers (*n* = 83480)			*P*
	*N* ^a^	(%)^b^	95%	CI	(%)^b^	95%	CI	(%)^b^	95%	CI	
Gender											‡
Boy	764506	51.82	51.74	51.90	50.52	50.44	50.60	73.82	73.75	73.89	
Girl	710755	48.18	48.10	48.26	49.48	49.40	49.56	26.18	26.11	26.25	
Grade											‡
7	212116	14.35	14.29	14.41	14.94	14.88	15.00	4.54	4.51	4.57	
8	226102	15.30	15.24	15.36	15.73	15.67	15.79	8.10	8.06	8.14	
9	239627	16.21	16.15	16.27	16.57	16.51	16.63	10.27	10.22	10.32	
10	276372	18.70	18.64	18.76	18.44	18.38	18.50	23.09	23.02	23.16	
11	271657	18.38	18.32	18.44	17.96	17.90	18.02	25.39	25.32	25.46	
12	251976	17.05	16.99	17.11	16.36	16.30	16.42	28.63	28.56	28.70	
Monthly allowance											‡
US$0	167157	11.35	11.30	11.40	11.77	11.72	11.82	4.46	4.43	4.49	
US$0.03–49.71	734396	49.89	49.81	49.97	51.51	51.43	51.59	22.82	22.75	22.89	
US$49.72–116.03	331284	22.50	22.43	22.57	22.34	22.27	22.41	25.25	25.18	25.32	
US$ ≧ 116.04	239326	16.26	16.20	16.32	14.39	14.33	14.45	47.47	47.39	47.55	
Parent educational level											‡
Junior high school and below	121374	8.90	8.85	8.95	8.42	8.37	8.47	17.06	17.00	17.12	
Senior high school	534386	39.18	39.10	39.26	38.63	38.55	38.71	48.63	48.55	48.71	
University or college	535077	39.24	39.16	39.32	39.99	39.91	40.07	26.27	26.20	26.34	
Graduate school	172918	12.68	12.62	12.74	12.95	12.89	13.01	8.04	7.99	8.09	
Smoking status of parents											‡
Both smoke	118445	8.19	8.15	8.23	7.37	7.33	7.41	22.27	22.20	22.34	
Only the father smokes	473158	32.71	32.63	32.79	32.30	32.22	32.38	39.77	39.69	39.85	
Only the mother smokes	28997	2.00	1.98	2.02	1.90	1.88	1.92	3.74	3.71	3.77	
Neither smokes	826062	57.10	57.02	57.18	58.42	58.34	58.50	34.22	34.14	34.30	
Smoking status of close friends											‡
Yes	619475	42.04	41.96	42.12	39.11	39.03	39.19	92.17	92.13	92.21	
No	854103	57.96	57.88	58.04	60.89	60.81	60.97	7.83	7.79	7.87	
Access to free cigarettes											‡
Yes	43428	2.98	2.95	3.01	2.51	2.48	2.54	11.22	11.17	11.27	
No	1416016	97.02	96.99	97.05	97.49	97.46	97.52	88.78	88.73	88.83	
Exposure to cigarette advertisements											‡
Yes	337111	22.89	22.82	22.96	22.28	22.21	22.35	33.24	33.16	33.32	
No	1135769	77.11	77.04	77.18	77.72	77.65	77.79	66.76	66.68	66.84	
Attended classes concerning the dangers of tobacco											‡
Yes	697677	56.01	55.92	56.10	57.03	56.94	57.12	39.23	39.14	39.32	
No	547990	43.99	43.90	44.08	42.97	42.88	43.06	60.77	60.68	60.86	

^a^The total numbers under each variable were not equal due to missing value(s).^b^Summed percentages in each column. P: Rao–Scott chi-square test was used to test the difference between independent variables and current cigarette smoking. ‡ indicates *p* values <0.001.

**Table 2:** Demographics of e-cigarette users and non-users among Taiwanese adolescents in 2018.

Characteristic	All (*N* = 1484052)				e-Cigarette non-users (*n* = 1443329)			e-Cigarette users (*n* = 40723)			*P*
	*N* ^a^	(%)^b^	95%	CI	(%)^b^	95%	CI	(%)^b^	95%	CI	
Gender											‡
Boy	769396	51.94	51.86	52.02	51.32	51.24	51.40	74.40	74.33	74.47	
Girl	711977	48.06	47.98	48.14	48.68	48.60	48.76	25.60	25.53	25.67	
Grade											‡
7	212325	14.31	14.25	14.36	14.5	14.44	14.56	7.35	7.31	7.39	
8	226778	15.28	15.22	15.34	15.37	15.31	15.43	12.01	11.96	12.06	
9	239127	16.11	16.05	16.17	16.21	16.15	16.27	12.69	12.64	12.74	
10	278851	18.79	18.73	18.85	18.8	18.74	18.86	18.28	18.22	18.34	
11	273690	18.44	18.38	18.50	18.29	18.23	18.35	23.97	23.90	24.04	
12	253280	17.07	17.01	17.13	16.82	16.76	16.88	25.69	25.62	25.76	
Monthly allowance											‡
US$0	168552	11.4	11.35	11.45	11.54	11.49	11.59	6.73	6.69	6.77	
US$0.03–49.71	737600	49.9	49.82	49.98	50.49	50.41	50.57	29.16	29.09	29.23	
US$49.72–116.03	332042	22.46	22.40	22.53	22.46	22.39	22.53	22.73	22.66	22.80	
US$ ≧ 116.04	239878	16.23	16.17	16.29	15.52	15.46	15.58	41.38	41.30	41.46	
Parent educational level											‡
Junior high school and below	121468	8.88	8.83	8.93	8.72	8.67	8.77	15.00	14.94	15.06	
Senior high school	536363	39.21	39.13	39.29	39.06	38.98	39.14	44.90	44.82	44.98	
University or college	536449	39.22	39.13	39.30	39.44	39.36	39.52	30.73	30.65	30.81	
Graduate school	173655	12.69	12.64	12.75	12.78	12.72	12.84	9.37	9.32	9.42	
Smoking status of parents											‡
Both smoke	119551	8.23	8.18	8.27	7.92	7.88	7.96	19.79	19.73	19.85	
Only the father smokes	475080	32.7	32.63	32.78	32.53	32.45	32.61	39.04	38.96	39.12	
Only the mother smokes	29250	2.01	1.99	2.04	1.95	1.93	1.97	4.28	4.25	4.31	
Neither smokes	828801	57.05	56.97	57.13	57.6	57.52	57.68	36.89	36.81	36.97	
Smoking status of close friends											‡
Yes	624300	42.17	42.09	42.25	41.13	41.05	41.21	80.12	80.06	80.18	
No	856066	57.83	57.75	57.91	58.87	58.79	58.95	19.88	19.82	19.94	
Access to free cigarettes											‡
Yes	45011	3.07	3.04	3.10	2.8	2.77	2.83	13.92	13.86	13.98	
No	1420031	96.93	96.90	96.96	97.2	97.17	97.23	86.08	86.02	86.14	
Exposure to cigarette advertisements											‡
Yes	338125	22.87	22.80	22.93	22.53	22.46	22.60	35.46	35.38	35.54	
No	1140642	77.13	77.07	77.20	77.47	77.40	77.54	64.54	64.46	64.62	
Attended classes concerning the dangers of tobacco											‡
Yes	697677	56.01	55.92	56.10	56.23	56.14	56.32	45.35	45.26	45.44	
No	547990	43.99	43.90	44.08	43.77	43.68	43.86	54.65	54.56	54.74	

^a^The total numbers under each variable were not equal due to missing value(s).^b^Summed percentages in each column. P: Rao–Scott chi-square test was used to test the difference between independent variables and current use of e-cigarettes. ‡ indicates *p* values <0.001.

**Table 3:** Demographics of IQOS users and non-users among Taiwanese adolescents in 2018.

Characteristic	All (*N* = 1501527)				IQOS non-users (*n* = 1466516)			IQOS users (*n* = 35011)			*P*
	*N* ^a^	(%)^b^	95%	CI	(%)^b^	95%	CI	(%)^b^	95%	CI	
Gender											‡
Boy	779780	52.03	51.95	52.11	51.64	51.56	51.72	68.96	68.89	69.03	
Girl	718800	47.97	47.89	48.05	48.36	48.28	48.44	31.04	30.97	31.11	
Grade											∗
7	214748	14.30	14.25	14.36	14.35	14.29	14.41	12.20	12.15	12.25	
8	229647	15.29	15.24	15.35	15.36	15.30	15.42	12.56	12.51	12.61	
9	241913	16.11	16.05	16.17	16.17	16.11	16.23	13.53	13.48	13.58	
10	281736	18.76	18.70	18.83	18.69	18.63	18.75	21.65	21.58	21.72	
11	277029	18.45	18.39	18.51	18.46	18.40	18.52	17.99	17.93	18.05	
12	256453	17.08	17.02	17.14	16.96	16.90	17.02	22.07	22.00	22.14	
Monthly allowance											‡
US$0	170113	11.38	11.32	11.43	11.40	11.35	11.45	10.49	10.44	10.54	
US$0.03–49.71	744969	49.82	49.74	49.90	50.25	50.17	50.33	31.70	31.63	31.77	
US$49.72–116.03	335925	22.46	22.40	22.53	22.47	22.40	22.54	22.24	22.17	22.31	
US$ ≧ 116.04	244405	16.34	16.28	16.40	15.89	15.83	15.95	35.57	35.49	35.65	
Parent educational level											‡
Junior high school and below	123637	8.93	8.89	8.98	8.80	8.75	8.85	14.36	14.30	14.42	
Senior high school	543645	39.28	39.20	39.36	39.12	39.04	39.20	45.78	45.70	45.86	
University or college	541726	39.14	39.06	39.22	39.39	39.31	39.47	28.51	28.43	28.59	
Graduate school	175088	12.65	12.59	12.71	12.68	12.62	12.74	11.35	11.30	11.40	
Smoking status of parents											‡
Both smoke	121901	8.29	8.25	8.34	8.08	8.04	8.12	17.72	17.66	17.78	
Only the father smokes	480892	32.72	32.64	32.79	32.70	32.62	32.78	33.46	33.38	33.54	
Only the mother smokes	29853	2.03	2.01	2.05	1.96	1.94	1.98	5.07	5.03	5.11	
Neither smokes	837168	56.96	56.88	57.04	57.26	57.18	57.34	43.75	43.67	43.83	
Smoking status of close friends											‡
Yes	633620	42.32	42.24	42.39	41.67	41.59	41.75	70.40	70.33	70.47	
No	863766	57.68	57.61	57.76	58.33	58.25	58.41	29.60	29.53	29.67	
Access to free cigarettes											‡
Yes	46068	3.11	3.08	3.14	2.82	2.79	2.85	16.75	16.69	16.81	
No	1435773	96.89	96.86	96.92	97.18	97.15	97.21	83.25	83.19	83.31	
Exposure to cigarette advertisements											‡
Yes	342874	22.92	22.85	22.99	22.74	22.67	22.81	30.94	30.87	31.01	
No	1153174	77.08	77.01	77.15	77.25	77.18	77.32	69.06	68.99	69.13	
Attended classes concerning the dangers of tobacco											
Yes	707812	55.95	55.86	56.03	55.99	55.90	56.08	53.90	53.81	53.99	
No	557325	44.05	43.97	44.14	44.01	43.92	44.10	46.10	46.01	46.19	

^a^The total numbers under each variable were not equal due to missing value(s).^b^Summed percentages in each column. P: Rao–Scott chi-square test was used to test the difference between independent variables and current use of IQOS. ^∗^ indicates *P* < 0.05 and ‡ indicates *P* < 0.001.

**Table 4:** Three logistic regression models illustrating factors correlated with current use of cigarettes, e-cigarettes, and IQOS.

Independent variable	*Y* = cigarette				*Y* = e-cigarette				*Y* = IQOS			
	aOR	95%	CI	*P*	aOR	95%	CI	*P*	aOR	95%	CI	*P*
Gender												
Boy	2.50	1.95	3.20	‡	2.36	1.88	2.96	‡	1.68	1.33	2.11	‡
Girl	1				1				1			
Grade												
7	1				1				1			
8	1.12	0.77	1.62		1.19	0.79	1.80		0.72	0.49	1.06	
9	1.48	1.00	2.21		0.97	0.64	1.47		0.68	0.46	1.00	∗
10	1.81	1.12	2.93	∗	0.90	0.61	1.33		0.82	0.58	1.15	
11	1.75	1.12	2.73	∗	1.18	0.75	1.86		0.52	0.35	0.76	‡
12	1.85	1.15	2.96	∗	1.10	0.75	1.62		0.64	0.43	0.95	∗
Monthly allowance												
US$0	1				1				1			
US$0.03–49.71	1.19	0.84	1.70		1.20	0.63	2.28		0.66	0.46	0.96	∗
US$49.72–116.03	1.91	1.38	2.66	‡	1.28	0.68	2.41		0.83	0.54	1.28	
US$ ≧ 116.04	3.95	2.81	5.56	‡	2.73	1.45	5.12	**†**	1.44	0.94	2.19	
Parent educational level												
Junior high school and below	1				1				1			
Senior high school	0.67	0.55	0.82	‡	0.85	0.63	1.14		0.80	0.60	1.08	
University or college	0.49	0.38	0.64	‡	0.75	0.51	1.08		0.65	0.48	0.90	**†**
Graduate school	0.59	0.35	1.00	∗	0.70	0.42	1.18		0.72	0.48	1.11	
Smoking status of parents												
Both smoke	2.57	1.97	3.34	‡	2.02	1.24	3.29	**†**	1.46	1.02	2.09	∗
Only the father smokes	1.43	1.21	1.68	‡	1.52	1.47	1.56	‡	0.95	0.72	1.25	
Only the mother smokes	1.38	0.93	2.03		1.51	0.86	2.68		1.30	0.68	2.48	
Neither smokes	1				1				1			
Smoking status of close friends												
Yes	11.44	8.91	14.68	‡	3.93	3.04	5.08	‡	2.76	2.15	3.54	‡
No	1				1				1			
Access to free cigarettes												
Yes	3.97	2.95	5.35	‡	3.49	2.48	4.90	‡	4.20	3.06	5.77	‡
No	1				1				1			
Exposure to cigarette advertisements												
Yes	1.27	1.10	1.46	‡	1.45	1.15	1.83	**†**	1.14	0.90	1.45	
No	1				1				1			
Attended classes concerning the dangers of tobacco												
Yes	0.59	0.51	0.69	‡	0.77	0.62	0.97	∗	1.02	0.83	1.25	
No	1				1				1			

aOR: adjusted odds ratios. ^∗^*P* < 0.05; †*P* < 0.01; ‡*P* < 0.001.

## Data Availability

The data used in this study were obtained from the Health Promotion Administration, Ministry of Health and Welfare, Taiwan. The authors have no right to disclose data and all of the authors signed confidentiality contracts. The authors only have the data, in which links to personal information were removed.
